# Methodological considerations regarding Joyce et al. 2016

**DOI:** 10.1186/s40345-017-0086-4

**Published:** 2017-05-31

**Authors:** Christopher Baethge, Leonardo Tondo, Ross J. Baldessarini

**Affiliations:** 1 Department of Psychiatry and Psychotherapy, University of Köln, Cologne, Germany; 2 Department of Psychiatry, Harvard Medical School, Boston, MA USA

Sir,

The recent review by Joyce et al. (2016) concludes that early intervention and treatment of bipolar disorder (BD) patients were consistently superior in 10 studies selected for analysis, largely involving short-term responses following an index episode of illness. We agree that there are compelling clinical reasons to encourage timely identification and treatment of such patients in efforts to limit long-term morbidity. However, we reported previously, based on 39 published reports plus original data from a large international sample, that neither latency from illness-onset nor the number of illness episodes before initiating treatment was related to long-term morbidity (percent of time ill or episodes/year) in BD subjects during 3.8–5.4 years of treatment (Baethge et al. 2003a; Bratti et al. 2003). Most of these studies were not included in the review by Joyce et al. (2016). We considered all subjects treated at various times or following various numbers of recurrences, and found that long-term morbidity following initial treatment did not vary significantly with treatment-delay or episode-count.

One possible explanation for the differences in conclusions arising in these studies may be caused by the lack of search terms more specific to the pertinent literature, such as “delay” (Baethge et al. 2003b) or “latency” (Baldessarini et al. 2003) in the overview by Joyce and colleagues.

Also important is a methodological problem in several studies referred to by Joyce et al.: comparing treatment response following early versus late interventions is likely to compare clinically dissimilar sub-populations. Early intervention groups include patients with a range of prognoses and illness severities, whereas later intervention involves subjects who have experienced illness recurrences and may have been exposed to various periods of active, failed, and discontinued treatment. That is, it seems likely that samples with a range of prognoses were compared to generally less favorable samples; such comparisons would be expected to yield more favorable initial treatment responses among early intervention subjects, as was found. As examples, this confounding factor pertains to studies by Franchini et al. (1999) and Keck et al. (1995).

Other methodological problems include use of multiple outcome measures not adjusted for multiple comparisons (e.g., Colom et al. 2010), as well as use of cut-off points selected retrospectively with risk of false-positive findings (e.g., Swann et al. 1999). The fact that studies used different treatments and that some investigated maintenance treatment whereas others focused on acute treatment introduces substantial heterogeneity and adds to the considerable difficulties in interpreting the results.

A powerful potential motivating factor favoring early intervention in BD would be a “progressive” course, in which more-or-less euthymic intervals usually become shorter with a growing number of illness recurrences. This hypothesis, while plausible at first sight, has been considered repeatedly over the past century. However, evidence supporting it has been inconsistent and generally unfavorable; moreover, potential sampling bias has been noted when subjects unmatched for recurrence counts are compared (Oepen et al. 2004; Baldessarini et al. 2012).

In conclusion, we note that patients treated early in the course of BD are likely to include a proportion with relatively favorable responses to treatment, and fewer favorable cases among those with a longer history of illness and variable responses to treatment. Our previous findings also support the impression that some morbidity in BD remains during long-term treatment but that it is not worse after longer delay of treatment or more previous illness recurrences. This conclusion runs counter to current expectations that early therapeutic intervention in BD might lead to long-term reductions of morbidity and disability superior to what can be achieved by later interventions. Again, this hypothesis is attractive, but it is far from undisputed (Duffy et al. 2016). On clinical and ethical grounds, we do strongly encourage timely recognition and appropriate treatment of BD patients in efforts to limit risks of morbidity, disability, and mortality which can arise from prolonged and inadequately treated illness. However, the hypotheses that BD is generally progressive and that early treatment intervention can modify its long-term course should be considered plausible but unproved at this point.
